# Surface modification of hollow magnetic Fe_3_O_4_@NH_2_-MIL-101(Fe) derived from metal-organic frameworks for enhanced selective removal of phosphates from aqueous solution

**DOI:** 10.1038/srep30651

**Published:** 2016-07-29

**Authors:** Yan Li, Qiying Xie, Qian Hu, Chengping Li, Zhangjie Huang, Xiangjun Yang, Hong Guo

**Affiliations:** 1 School of Chemistry Science and Engineering, Yunnan University, Kunming 650091, Yunnan, China; 2 Yunnan Key Laboratory of Micro/Nano Materials & Technology, Kunming 650091, Yunnan, China

## Abstract

Hollow magnetic Fe_3_O_4_@NH_2_-MIL-101(Fe) derived from metal-organic frameworks are fabricated through a general facile strategy. The synthetic parameters are regulated to control the shape of the as-prepared samples. The concentration of phosphates decreased sharply from the initial 0.60 to 0.045 mg.L^−1^ with the exposure time in 50 minutes. The correlation between the most significant parameters such as contact time, adsorbent dose, pH, as well as adsorption capacities was optimized, and the effects of these parameters on the removal efficiency of phosphates were investigated. Surface functionalization of magnetic hollow materials is a well-designed way to bridge the gap between high adsorption activity, excellent separation and recovery of phosphates from the water treatment system. Therefore, it exhibits a remarkable selective removal of phosphates from aqueous solution.

Phosphates are widely used for various industries such as food, agriculture, beverage and detergent, and thus these phosphate-containing products led to large discharge of excess phosphates, causing rapid growth of aquatic plants and algae in the water. Particularly, eutrophication worsens water quality and disturbs the balance of aquatic ecology. Undoubtedly, it has become a global necessity to efficiently decontaminate phosphates with minimal environmental impact. To date, the normal treatment processes of phosphates from aqueous solutions contain enzymatic biodegradation^1^, adsorption^2^, electrochemistry^3^, precipitation and flotation^4^, of which removal of phosphates by adsorption techniques is the most employed competitive routine owning to its environmental process, simple and fast operation with low cost^5^,^6^. Therefore, much effort is focused on the developing new adsorbents with high adsorption capacities.

Over the past few decades, metal-organic frameworks as a new class of organic-inorganic hybrid functional materials with high porosity, large surface area and morphology can be easily tuned upon selection of different metal ions and organic bridging ligands^7^,^8^,^9^, have been developed as a new class of solid adsorbents. Compared with conventional solid adsorbents such as mesoporous silica materials^10^,^11^ and activated carbon^12^,^13^, MOFs exhibit more virtues of versatile framework compositions and exposed active sites, tunable pore sizes, large specific surface. Large amounts of MOFs are proved to be kept stable and show good adsorption ability for the removal of pollutants from aqueous solution such as heave metal ions^14^, organic dye^15^,^16^, phenols^17^, oil droplets^18^ and humic acid^19^. Nevertheless, to our best knowledge, few studies have been reported to investigate the removal of phosphate from water by MOFs via the adsorption, because this process not only needs adequate pore size of MOFs but also specific active sites. Only most recently, J. L. Gu and coworkers^20^ fabricated Zr-based MOFs of UiO-67 with effective adsorption and enhanced removal of organophosphorus pesticides from aqueous solution. K.Y.A. Lin and coworkers^21^ reported zirconium-based metal organic frameworks, which expressed highly selective adsorbents for removal of phosphate from water and urine. These results prompt us to carry out the corresponding work on removal of phosphates from wastewater by MOFs. However, in fact these researches are most concentrated on Zr-based materials with high cost. Therefore, it is also great desire that designing and constructing new structures with low cost such as Fe-based MOFs with exceptional stability.

Another popular efficient and innovating solution to environmental problems has been focused on controlling crystal phase, particle size, crystallinity, morphology of materials. Nanomaterials are one of the most reactive minerals that have high surface area, strong oxidizing/adsorptive abilities, and good stability. Especially, the hollow structures at the nano scale are attracting fast growing interest because they can be used to find new applications owing to their specific geometry and structural flexibility. For example, our previous synthesized hybrid hollow ring-shaped Bi_2_WO_6_@CeO_2_ hybrid nanoparticle aggregates exhibited a remarkable photocatalytic detoxification of cyanide and degradation of dye under visible light^22^ and prepared Nano MnO_2_ nanofilm can serve as an ideal candidate for heavy metal ions removal in water treatment^23^. Up to now, the common synthetic strategy for the fabrication of those structures with complex interiors employs sacrificial templates, such as polymer, silica, carbon inorganic spheres and ionic liquids. However, templating methods for constructing complex nanostructures are usually time consuming and costly because of the need for the synthesis of the templates and the multi-step templating process, and thus it is highly desirable to develop facile, scalable approaches for the rational synthesis of hollow structures with designed interior structures.

Futhermore, it is can be noticed that the current adsorbents of phosphates are not easy to handle and difficult to separate, which need gravitational sedimentation with long time or extra mechanical separation such as centrifuge resulting of high operational cost. The elaboration of magnetic microspheres tremendous interest owing to the versatility of the collective functionalities and their numerous potentialities in the application scope of environment treatment. In fact, magnetite is an ideal oxide support, easy to prepare, having a very active surface for adsorptions or immobilization of metals and ligands, which can be separated by magnetic decantation after the reaction, thus making it a more sustainable adsorbents. In the last few years, various forms of iron oxides such as FeO (wustite) and Fe_2_O_3_ (iron III oxides) were successfully deployed in catalysis and envirenment^24^,^25^,^26^. Additionally, the incorporation of metal nanoparticles into metal-organic frameworks (MOFs) used as adsorbents also attracts much attention in water treatment. Though these procedures are effective, each designed strategy alone always leads to limited improvement in the adsorption and separated performance of phosphate from aqueous solution. And thus, the development of a facile, scalable and controllable fabrication of durable hybrid hollow magnetic materials with satisfactory adsorption activity, easily separated and excellent selectivity is still highly desirable. Besides, to our best knowledge, reports on the fast synthesis of hollow magnetic Fe_3_O_4_ with metal-organic frameworks are quite rare compared with current methods that produced nanostructure, and can be an advantage for chemists to elaborate possible new constructions from all chemical components without any time-restricted conditions.

Herein, we chose Fe_3_O_4_@NH_2_-MIL-101(Fe) nanostructures to demonstrate our concept and propose a general strategy to prepare hollow porous magnetic MOFs as Fig. 1. The as-prepared hollow Fe_3_O_4_ is hired as the template, and then the NH_2_-MIL-101(Fe) is coated in the surface of Fe_3_O_4_ uniformly. In this work, the advantage of novel hollow structures, the virtues of MOFs and magnetic recover properties are well integrated to solve the problems in the separation and recovery of phosphates from water. As a result, these magnetic adsorbents are anticipated to high adsorption activity as well as good separation and recollection rates from the aqueous solution.

## Results and Discussion

### Structure and morphology of hollow magnetic Fe_3_O_4_@NH_2_-MIL-101(Fe)

**Figure 2:**
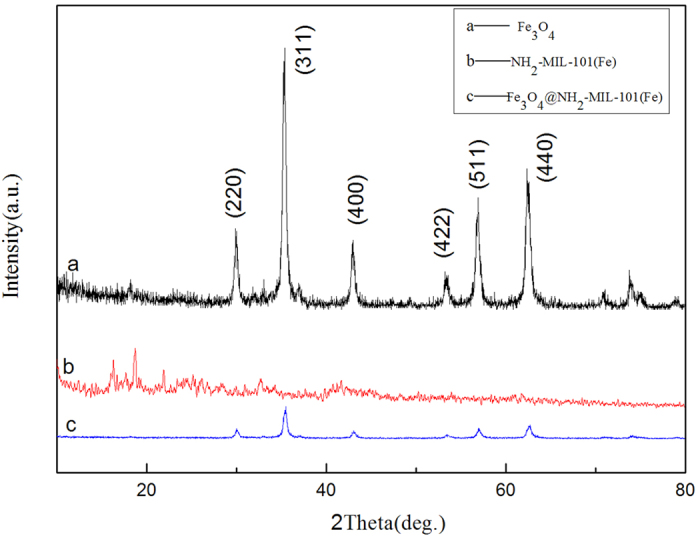
X-ay diffraction (XRD) patterns of (**a**) Fe_3_O_4_, (**b**) NH_2_-MIL-101(Fe) and (**c**) Fe_3_O_4_@NH_2_-MIL-101(Fe).

**Figure 3:**
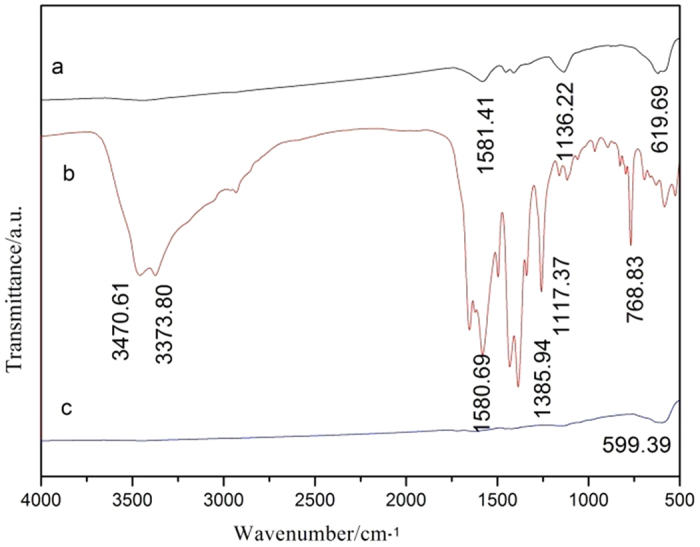
FTIR spectra of prepared (**a**) Fe_3_O_4_, (**b**) NH_2_-MIL-101(Fe) and (**c**) Fe_3_O_4_@NH_2-_MIL-101(Fe).

The crystallographic structure and phase purity of Fe_3_O_4_ precursor, NH_2_-MIL-101(Fe) and hollow Fe_3_O_4_@NH_2_-MIL-101(Fe) magnetic sample were analyzed by XRD, as shown in Fig. 2. All the diffraction peaks can be indexed to the Fe_3_O_4_ (JCPDS card no. 19-0629). No other impurity peaks are observed, indicating a complete thermal conversion of the MOF precursors into Fe_3_O_4_ nanostructures. Detailed analysis of the peak broadening of the (3 1 1) reflection of Fe_3_O_4_@NH_2_-MIL-101(Fe) using the Scherrer equation indicates an average crystallite size ca. 4 nm, suggesting that these particles are composed of nanocrystal subunits. The FTIR spectrum images of the prepared hollow structured Fe_3_O_4_@NH_2_-MIL-101(Fe) magnetic sample, NH_2_-MIL-101(Fe) and its Fe_3_O_4_ precursor are shown in Fig. 3. The broad absorption peaks from 3432 to 2980 cm^−1^ are associated with the stretching vibrations of the -OH group of absorbed water molecules and absorption of CO_2_ in the air, and the peak of 1581 cm^−1^ is assigned to the bending vibrations of the water molecules. For the Fe_3_O_4_@NH_2_-MIL-101(Fe), the spikes from 1500 to 1000 cm^−1^ are assigned to the asymmetric and symmetric stretching vibrations of the carbonyl group of organics. The strongest broad peaks in the range of 400-1000 cm^−1^ are contribution from the face-centered cubic phase Fe-O. The peak intensity of Fe-O bond for final products is different from that of precursor, implying the structure of prepared sample has some discrepancy with its precursor.

**Figure 4:**
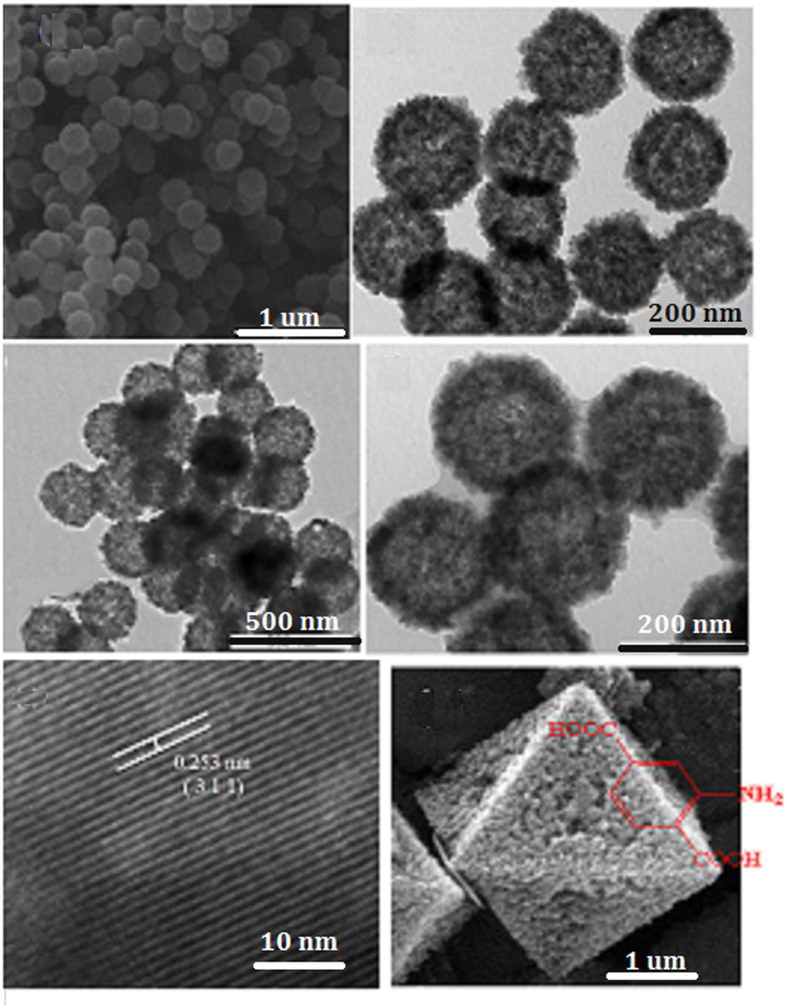
SEM (**a**) and TEM (**b**) images of hollow Fe_3_O_4_ precursor. TEM (**c,d**) and HRTEM (**e**) micrographsthe of as-prepared hollow porous magnetic Fe_3_O_4_@NH_2_-MIL-101(Fe). SEM (**f**) morphology of the prepared NH_2_-MIL-101(Fe).

The SEM and TEM images of the prepared hollow Fe_3_O_4_ precursors are shown as Fig. 4a,b. It is obvious that the precursors are hollow sub-microsphere uniformly with average diameter of ca. 300 nm according to Fig. 4a. TEM (Fig. 4b) reveals that these sub-microspheres exhibit hollow structures obviously. The unique hollow porous morphologies of Fe_3_O_4_@NH_2_-MIL-101(Fe) magnetic products are characterized by TEM and HR-TEM, as illustrated in Fig. 4c–e. Figure 4c shows the as-prepared samples keeps the original morphology of its precursors. The TEM images in Fig. 4c and d show the hollow structures of the products, exhibiting a visible hollow interior structure obviously. Especially, a typical structure with well-defined interior and shell can also be detected and the thickness of shell of samples is ca. 150–200 nm. The surface of samples exhibits porous frame, which hierarchical structure is resulted from MOFs. Furthermore, the surfaces coat the layers of NH_2_-MIL-101(Fe) obviously according to Fig. 4d. The size of as-synthesized Fe_3_O_4_-MOFs are much smaller than those reported by Zhu very most recently^27^. The lattice fringe is observed obviously, and the lattice spacing (0.253 nm) agrees with Fe_3_O_4_ (3 1 1) plane spacing from Fig. 4e. The morphology of NH_2_-MIL-101(Fe) (Fig. 4f) show a octahedral structure, which is similar with the Zr-based materials^20^,^21^.

**Figure 5:**
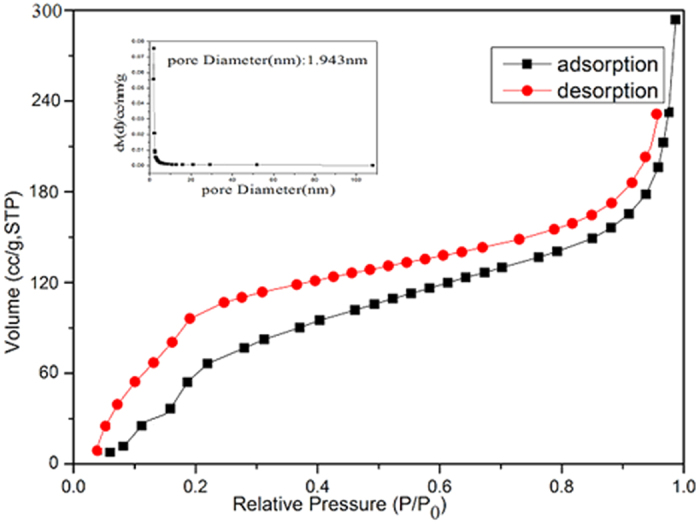
N_2_ adsorption/desorption isotherm (77K) curve for hollow porous magnetic Fe_3_O_4_@NH_2_-MIL-101(Fe).

The N_2_ adsorption/desorption isotherms and the pore size distribution of the obtained hollow porous hollow Fe_3_O_4_@NH_2_-MIL-101 (Fe) magnetic products are shown as Fig. 5, and their isotherms of Fe_3_O_4_ and pristine NH_2_-MIL-101 are shown in Fig. S1 (seeing the supporting information). The isotherms are identified as type IV, which are the characteristic isotherm of mesoporous materials. The pore size distribution data indicates that average pore diameters of the product are in the range of 1–2 nm. The BET surface area of the sample is 825.15 m^2^ g^−1^. Though this value is lower than those of MIL-100(Fe) obtained by the solvothermal method due to the nonporous Fe_3_O_4_ hells in the hollow magnetic materials, remarkably, the specific surface area of hollow porous magnetic Fe_3_O_4_@NH_2_-MIL-101(Fe) is far higher than most of the previous reported many other magnetic porous materials^28^,^29^.

**Figure 6:**
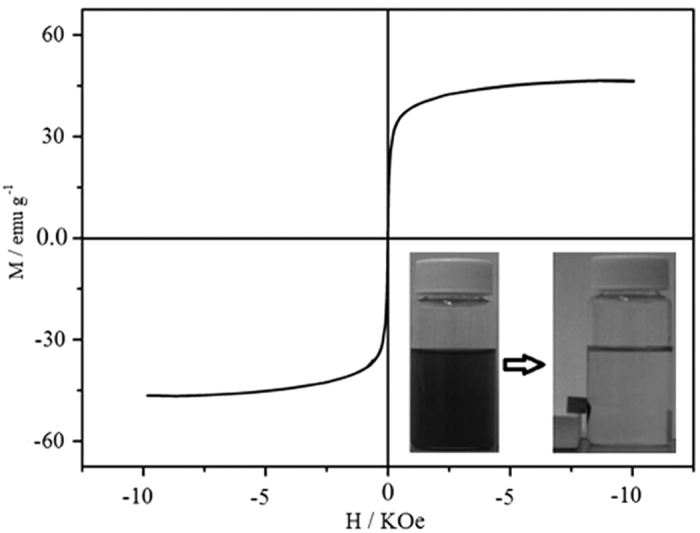
The magnetic hysteresis loop of hollow Fe_3_O_4_@NH_2_-MIL-101(Fe) magnetic products.

The magnetic properties of as-synthesized samples are measured on SQUID at room temperature in the applied field sweeping from-10 K to 10 K Oe. The curves show no remnant magnetization or coercivity, indicating the samples are expected magnetic behavior at room temperature. This depicts that no magnetization remained when the applied magnetic field is removed. The Ms (saturation magnetization) is about 45 emu g^−1^, suggesting a high magnetite content in the prepared hollow magnetic products, which is in agreement with the data from the literature^30^. The difference may come from the dipolar interactions among the NPs, the different particle sizes and the characters of the surfactant. The inset in Fig. 6 shows photographs of the hollow magnetic products dispersion and the response of these samples under an external magnetic force. The products are dispersed in ethanol with a concentration of 0.5 mg mL^−1^ by sonicating for several minutes. The photograph of the dispersion shows a light black nanoparticle solution. As an external magnetic field was applied, the particles are attracted by the magnet, leaving the ethanol solution clear and transparent. Removing the external magnetic field and sonicating the solution redispersed the particles into the solution, and the dispersion could be stable for more than 50 min. The attraction and redispersion processes can be readily altered by applying and removing an external magnetic field, showing great potential for the excellent separability of the hollow porous magnetic Fe_3_O_4_@NH_2_-MIL-101(Fe) in the liquid-phase reactants and products.

### Adsorption capacity

#### Effect of the adsorbent dosage on the phosphate adsorption

**Figure 7:**
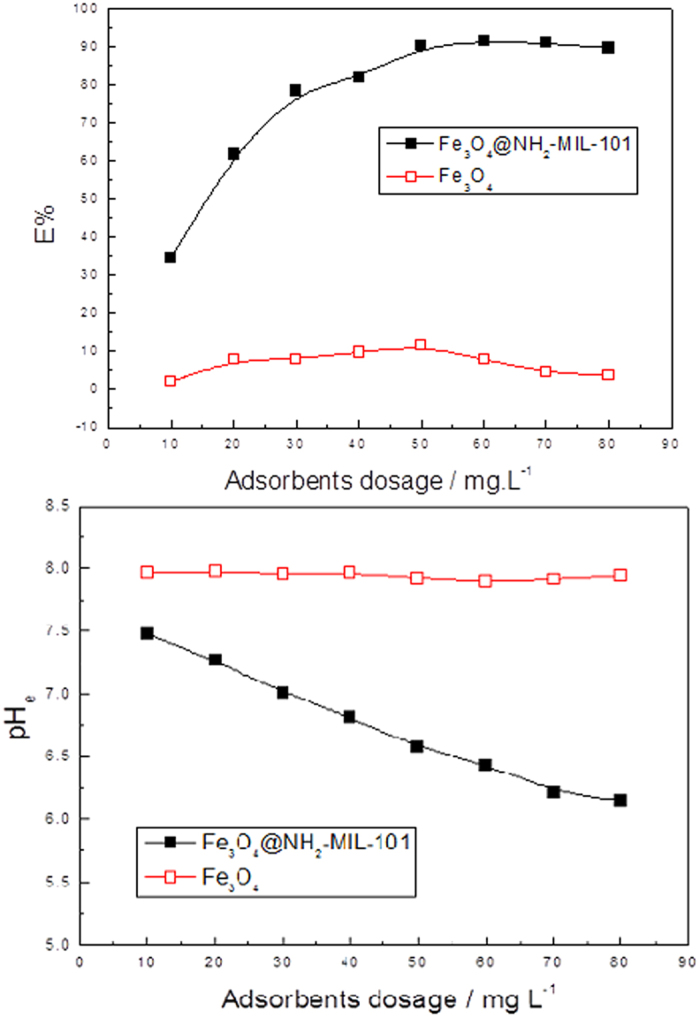
(**a**) Effects of the adsorbent dosage on the removal efficiency E%. (**b**) pH_e_ by Fe_3_O_4_@NH_2_-MIL-101(Fe). (Fe_3_O_4_@NH_2_-MIL-101(Fe), 2–16 mg; NaH_2_PO_4_ c_0_ = 0.6 mgL^−1^; V = 0.2 L; T = 293 K).

The most current reported adsorbents were focused on the water treatment in the system with high concentration even more than 50 mg L^−1^. However, rare reports developed researches on low concentrations. In fact, the concentrations of phosphate of most natural lake with eutrophication are little extra 1 mg L^−1^, and thus it is much desirable to design and construct novel adsorbents for low concentration, which will express particle value for water treatment. So, phosphate solution with C_0_ = 0.6 mg.L^−1^ is adopted for the test of adsorption capacities. The effect of the adsorbents dosage on the phosphate adsorption to Fe_3_O_4_@NH_2_-MIL-101(Fe) and Fe_3_O_4_ shown in Fig. 7a. When the adsorbent dosage of Fe_3_O_4_@NH_2_-MIL-101(Fe) increased from 10 to 60 mg.L^−1^. At the high adsorbent dosage, the total amount of adsorption sites was significantly greater and therefore a higher amount of phosphate was removed from water. Nevertheless, the removal efficiency was declined when the adsorbent dosage was higher than 70 mg.L^−1^. Furthermore, the Equilibrium pH value (pH_e_) had a drop tendency (Fig. 7b). For Fe_3_O_4_, the phosphate removal efficiency is very low, and the equilibrium pH value does not change. Therefore, the main absorption of phosphorus in the composite material is NH_2_-MIL-101(Fe). Thus, the Fe_3_O_4_@NH_2_-MIL-101(Fe) adsorbent dosage 60mg.L^−1^ was chosen for the rest of the adsorption experiments in this study. The phosphate removal efficiency increased from 34.50% to 92.33% corresponding to the concentration as low as 0.045 mg L^−1^ in 50 minutes, which value is far more than the two-class standard of China 0.1 mg.L^−1^, implying the hollow porous magnetic Fe_3_O_4_@NH_2_-MIL-101(Fe) materials having high adsorption efficiency.

#### Effect of pH on the phosphate adsorption

**Figure 8:**
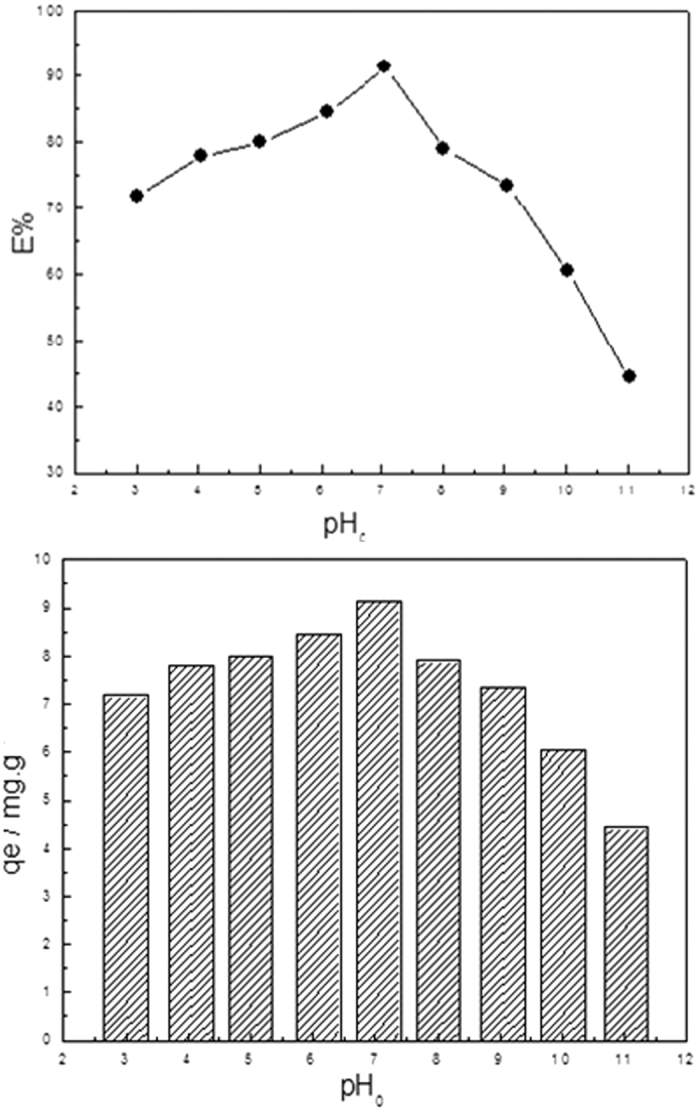
(**a**) Effects of the pH on the phosphate removal efficiency E%. (**b**) adsorption capacity q_e_ to Fe_3_O_4_@NH_2_-MIL-101(Fe). (Fe_3_O_4_@NH_2_-MIL-101(Fe), 12 mg; NaH_2_PO_4_ c_0_ = 0.6 mgL^−1^; V = 0.2 L; T = 293 K).

pH value of the solution is one of the main factors that affect the adsorption capacity of the adsorbent, not only for the presence form of adsorbate phosphate in the solution but also for the surface charge of the adsorbent^31^. Therefore, it is important to study the influence of pH value of the solutions during the adsorption process. Figure 8 shows the effects of the solution pH on the phosphate removal efficiency E% and adsorption capacity to Fe_3_O_4_@NH_2_-MIL-101(Fe). It is clear that when solution initial pH is in 3, the removal efficiency and adsorption capacity increase with the increase of pH, with the E% and q_e_ reaching the maximum values when solution initial pH is 7. After that, the removal efficiency and adsorption capacity decreases gradually. The effect of pH on the phosphate removal efficiency E% and adsorption capacity q_e_ to Fe_3_O_4_@NH_2_-MIL-101(Fe) exist two main reasons. Firstly, pH affects the existence form of phosphate in solution. Dissociation equilibrium of phosphate in aqueous solution are shown as equation 1^32^.


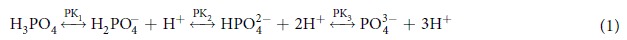


pK_1_ = 2.l5, pK_2_ = 7.20, pK_3_ = 12.33. When pH < 2.15, the phosphate in the solution present in the form of H_3_PO_4_, which is difficult to be adsorbed by the adsorbent. When 2.15 < pH < 7.2, the existence form of phosphate is mainly as 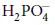 and the affinity of adsorption site is strong, so it is easy to be absorbed by the adsorbent. When pH > 7.2, the main form of phosphate in the solution was 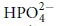 the affinity of adsorption sites are relatively weak, and thus the removal efficiency E% and adsorption capacity q_e_ shows a decreasing trend. Additionally, it is also noticed that pH affects the surface charge of the adsorbents Fe_3_O_4_@NH_2_-MIL-101(Fe). Under the acidic condition, the -NH_2_ in the adsorbent is easy to accept the hydrogen proton and forms a positively charged 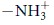. Under the strong electrostatic attraction, the adsorbate 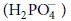 is easy to be adsorbed by Fe_3_O_4_@NH_2_-MIL-101(Fe)^33^,^34^. While the -NH_2_ on the adsorbent is deprotonated with increasing the concentration of OH^−^ under the alkaline conditions. And thus, it is not favorable for the adsorption process due to the electrostatic repulsion between the adsorbent and the adsorbate. Moreover, excessive OH^−^ and 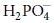 competes for adsorption sites, resulting in a decrease in the removal efficiency and adsorption capacity.

#### Effects of mixing time and Kinetics study on the phosphate adsorption

**Figure 9:**
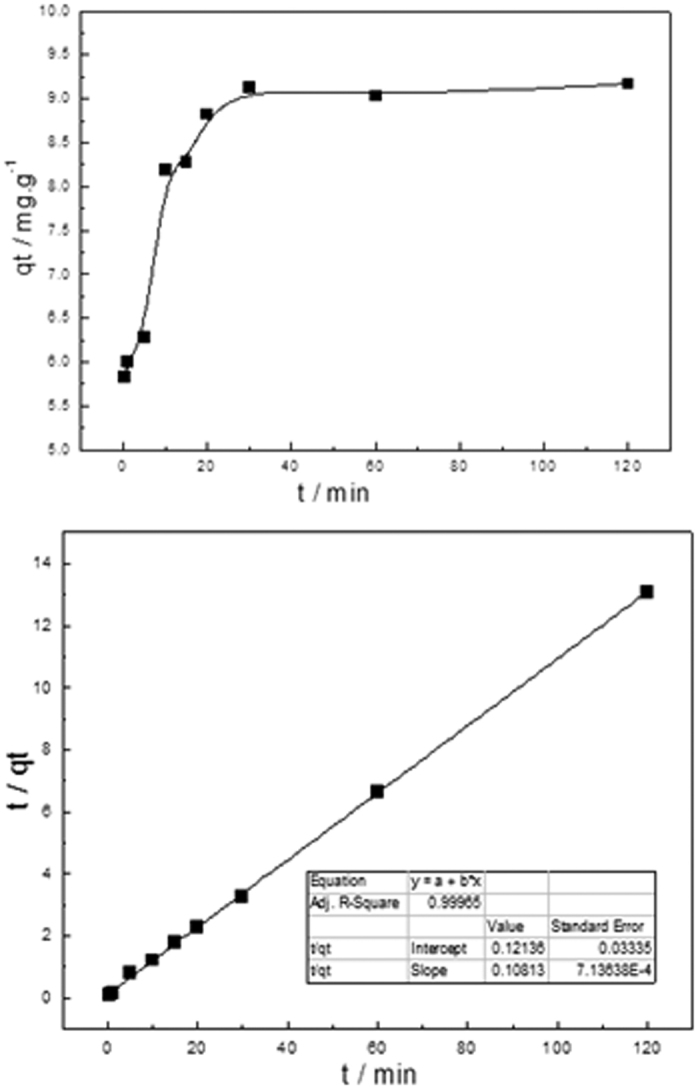
(**a**) Effects of the mixing time on the phosphate adsorption capacities (**a**) and the pseudo second order kinetic equation fitting line of phosphate adsorption. (**b**) Where k_2_ (gmg^−1^min^−1^) is the pseudo second order rate constant. (Fe_3_O_4_@NH_2_-MIL-101(Fe), 12 mg; NaH_2_PO_4_ c_0_ = 0.6 mgL^−1^; V = 0.2 L; T = 293 K).

It can be seen that the adsorption capacity increased rapidly in the beginning of the adsorption process and then approached a constant value (in Fig. 9a). In the first 20 min, the adsorption capacity of Fe_3_O_4_@NH_2_-MIL-101(Fe) had reached 8.82 mg.g^−1^, revealing the quick adsorption of phosphate to Fe_3_O_4_@NH_2_-MIL-101(Fe). It is interesting that the time of adsorption only need 30 minutes, which is much shorter than other reports. To quantitatively analyze the kinetics of the phosphate adsorption, plots of q_t_ versus t were further analyzed using the pseudo second order rate laws. The pseudo second order rate law was then used to analyze the kinetics as equation 2^35^, where k_2_ (g.mg^−1^.min^−1^) is the pseudo second order rate constant.


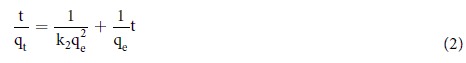


The fitting of the kinetic data was represented in Fig. 9b, showing the kinetic data points were well fitted. Correlation coefficients R_2_ = 0.99965, implying the pseudo second order equation was suitable model for the adsorption kinetics of Fe_3_O_4_@NH_2_-MIL-101(Fe). The estimated q_e_ is 9.25 mg.g^−1^ from the fitting lines, which was nearly consistent with the experimental data of 9.11 mg.g^−1^.

#### Effects of initial concentration and Adsorption isotherm of phosphate to Fe_3_O_4_@NH_2_-MIL-101(Fe)

To further analyze the adsorption isotherm data, the Langmuir isotherms was employed. The Langmuir model assumes that the adsorption occurs as a mono-layer on a homogenous surface where the number of adsorptive sites is finite. Once the adsorptive sites are occupied, they cannot adsorb other adsorbates. Therefore, a maximal adsorption capacity (q_max_) is expected in the Langmuir model which can be described as equation 3^36^.


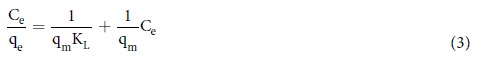


where K_L_ (L.mg^−1^) represents the Langmuir adsorption constant, associated with the adsorption bonding energy. Furthermore, the separation constant R_L_ is another important property of the Langmuir adsorption isotherm, which is used to characterize the degree of adsorption reaction carried out, and the expression is as equation 4^37^.


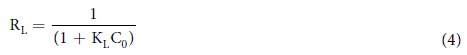


In low initial concentration of phosphorus solution (c_0_ = 0.1–1.0 mg.L^−1^), the isotherm data of phosphate adsorption to Fe_3_O_4_@NH_2_-MIL-101(Fe) fitted by the Langmuir model are shown in Fig. S2. Correlation coefficients of the fitting R_2_ = 0.99289. The estimated q_max_ of Fe_3_O_4_@NH_2_-MIL-101(Fe) from the fitting lines was 11.95 mgg^−1^, which was nearly consistent with the experimental data of q_e_ (10.92 mgg^−1^). K_L_ = 43.14 Lmg^−1^, which indicates that the Langmuir isotherm was suitable for describing the phosphate adsorption behavior of Fe_3_O_4_@NH_2_-MIL-101(Fe), and this adsorption behavior belongs to monolayer adsorption. As shown in Fig. S3, R_L_ decreased with the increase of the initial concentration of phosphorus solution, indicating that increasing the concentration of adsorbed substance is more beneficial to the adsorption^36^.

#### Effects of co-existing anions and magnetic adsorption performance of Fe_3_O_4_@NH_2_-MIL-101(Fe)

**Figure 10:**
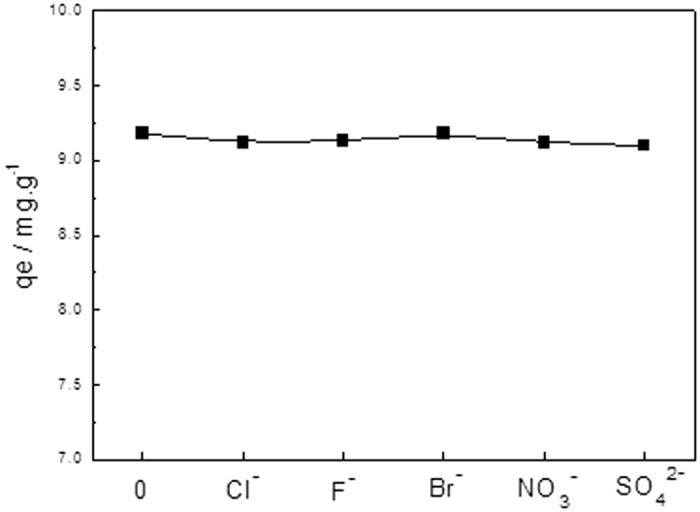
Effects of the Co-existing anions on the phosphate adsorption to Fe_3_O_4_@NH_2_-MIL-101(Fe). (Fe_3_O_4_@NH_2_-MIL-101(Fe), 12 mg; NaH_2_PO_4_ c_0_ = 0.6 mgL^−1^; V = 0.2 L; T = 293 K).

Since eutrophic water also contains other anions, it is important to examine the competing adsorption between phosphate and other anions to Fe_3_O_4_@NH_2_-MIL-101(Fe). Figure 10 reveals that even though the other anions are present in the phosphate solution and c_0_ of each ion species is 0.6 mgL^−1^, q_e_ of Fe_3_O_4_@NH_2_-MIL-101(Fe) remained almost unchanged. This indicates that the presence of chloride, fluorine, bromide, nitrate and sulfate anions did not hinder the adsorption of phosphate to Fe_3_O_4_@NH_2_-MIL-101(Fe). The adsorption of above mentioned anions to adsorbents was negligible, showing the prepared Fe_3_O_4_@NH_2_-MIL-101(Fe) possessed a high selectivity towards phosphate over the other anions. Moreover, the unique magnetism of the synthesized adsorbents contributes greatly to the fast collection of the disposals after adoption as Fig. S4, which exhibits excellent potential in the process of water treatment. Additionally, Figs S1–S4 and thermodynamic study on the phosphate adsorption are listed in the supplementary information.

Compared with the previous reported adsorbents^15^,^16^,^17^,^18^,^20^,^21^, the material reported here is very attractive due to its facile, fast, and improved adsorption activity. Surface functionalization of magnetic hollow materials is a well-designed way to bridge the gap between high adsorption activity and excellent separation and recovery from the water treatment system. Therefore, these functionalized magnetically adsorbents are increasingly being used in environment, catalysis, green chemistry and pharmaceutically significant reactions.

## Conclusions

In summary, hollow porous magnetic Fe_3_O_4_@NH_2_-MIL-101(Fe) materials are successfully synthesized by a facile and fast benign procedure. They exhibit excellent adsorption performance for enhanced selective removal of phosphates from aqueous solution. Moreover, the adsorbents can be easily separated from the reaction system. Compared to other adsorbents, the prepared materials are good recovery application and high adsorption activity due to the unique hollow structure, surface functionalization and good magnetic property. This general strategy may shed light on a novel avenue for the effective synthesis of hollow porous magnetic MOFs for environmental remediation, energy storage, drug delivery, catalyst and other new applications.

## Experimental Section

### Materials and Methods

#### Synthesis of hollow Fe_3_O_4_ precursor

Synthesis of hollow Fe_3_O_4_ is according to the improved method^38^. All chemicals were of analytical grade and used without further purification. Typically, FeCl_3_·6H_2_O (1.52 g), sodium citrate (3.02 g), urea (0.55 g) were dissolved in 80 mL deionized water and stirred for 30 min to form a homogeneous solution. Then 0.8 g PVP (MW30000) was added to the above solution with a continual stirring for 60 min. Subsequently, the light yellow solution was transferred into a 100 mL Teflon-lined stainless steel autoclave and held at 180 °C for 12 h. Finally, the black products were harvested through several rinse-centrifugation cycles with deionized water and absolute ethanol, and then dried at 60 °C under vacuum condition overnight.

#### Synthesis of hierarchical hollow Fe_3_O_4_@NH_2_-MIL-101(Fe) spheres

The controllable fabrication of hollow Fe_3_O_4_@NH_2_-MIL-101(Fe) nanostructures was used a versatile step-by-step assembly procedure. The typical procedure was as follows: 0.5 g Fe_3_O_4_ was dispersed in 40 mL of FeCl_3_·6H_2_O ethanol solution (10 mM) under ultrasound for 15 minutes, Then in 2-aminoterephthalic acid ethanol solution (40 mL,10 mM) for 4 h at 70 °C. During the interval the particles were separated by centrifugation and washed with ethanol, The final Fe_3_O_4_@NH_2_-MIL-101(Fe) nanostructures were obtained after amount of cycles, the samples were recovered by centrifugation and washed with ethanol, then dried in a vacuum at 50 °C for 8 hours.

#### Characterization

X-ray diffraction (XRD) was carried out to identify the phase composition of synthesized samples over the 2θ range from 3° to 90° using a Rigaku D/max-A diffractometer with Co Kα radiation. A Fourier transform infrared spectroscope (FTIR, Themo Nicolet 670FT-IR) was used for recording the FTIR spectra of the sample ranging from 400 to 4000 cm^−1^. Morphologies of the synthesized samples were observed with a AMRAY 1000B scanning electron microscope (SEM), and the microstructural characteristics of samples were observed by high-resolution transmission electron microscope (HR-TEM, JEOL JEM-2010) working at 200 kV accelerating voltage and the lattice structure was identified by selected area electron diffraction (SAED) technique. Nitrogen adsorption-desorption measurements were conducted at 77 K on a Micromeritics Tristar apparatus. Specific surface areas were determined following the Brunauer-Emmet-Teller analysis. The magnetization curve was measured at room temperature under a varying magnetic field from -10000 to 10000 Oe using a BHV-55 vibrating sample magnetometer (VSM).

#### Adsorption of phosphate to Fe_3_O_4_@NH_2_-MIL-101(Fe)

Adsorption behaviors of phosphate to Fe_3_O_4_@NH_2_-MIL-101(Fe) were studied using batch-type adsorption experiments. A certain amount of Fe_3_O_4_@NH_2_-MIL-101(Fe) was added to 0.2 L of phosphate solution with a given initial concentration (C_0_). The resulting mixture was then placed in a temperature-controllable magnetic stirrer at 200 rpm to begin the adsorption. The adsorption experiments were performed for 30 min to ensure that the adsorption reached the equilibrium. The adsorption capacity at equilibrium was denoted as *q*_e_ (mg g^−1^) was calculated using the following equation 5:


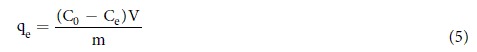


The phosphate removal efficiency (E%) of samples was calculated using the following equation 6:


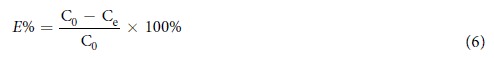


where m (g) is the weight of samples, *c*_e_ (mg.L^−1^) is equilibrium concentration of phosphorus and *V* represents the total volume of solution. The concentration of phosphate in supernatant was analyzed by National standard method of China (GB11893-89): Water quality-Determination of total phosphorus-Ammonium molybdate spectrophotometric method. In this study, the effect of the adsorbent dosage was first studied by varying adsorbent dosage from 10 to 80 mg L^−1^. In addition, the adsorption kinetics of phosphate was determined by mixing 12mg of samples with a 0.2 L of phosphate solution with C_0_ = 0.6 mg.L^−1^ at normal atmospheric temperature. The adsorption isotherm, on the other hand, was measured by fixing the amount of Fe_3_O_4_@NH_2_-MIL-101(Fe) (i.e., 12mg) while C_0_ was varied from 0.1 to 10 mg.L^−1^.

## Additional Information

**How to cite this article**: Xie, Q. *et al.* Surface modification of hollow magnetic Fe_3_O_4_@NH_2_-MIL-101(Fe) derived from metal-organic frameworks for enhanced selective removal of phosphates from aqueous solution. *Sci. Rep.* **6**, 30651; doi: 10.1038/srep30651 (2016).

## Supplementary Material

Supplementary Information

## Figures and Tables

**Figure 1:**
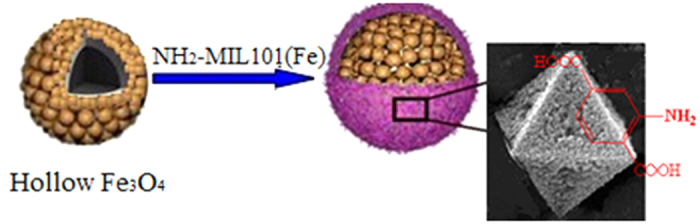
Representative illustration of the formation of hollow porous magnetic Fe_3_O_4_@NH_2_-MIL-101(Fe).
