# Supplementary material for: Surface modification of hollow magnetic Fe3O4@NH2-MIL-101(Fe) derived from metal-organic frameworks for enhanced selective removal of phosphates from aqueous solution
Source: Sci Rep. 2016 Jul 29;6:30651. doi: 10.1038/srep30651 (PMC4965783; doi:10.1038/srep30651)
Supplement: Supplementary Information [file srep30651-s1.doc]

Supporting information

**Surface modification of hollow magnetic Fe_3_O_4_@NH_2_-MIL-101(Fe) derived from metal-organic frameworks for enhanced selective removal of phosphates from aqueous solution**

Qiying Xie, Yan Li, Qian Hu, Chengping Li, Zhangjie Huang, Xiangjun Yang*, Hong Guo*[[1]](#footnote-2)*

*School of Chemistry Science and Engineering, Yunnan University, Kunming 650091, Yunnan, China*

**Electronic Supplementary Information**


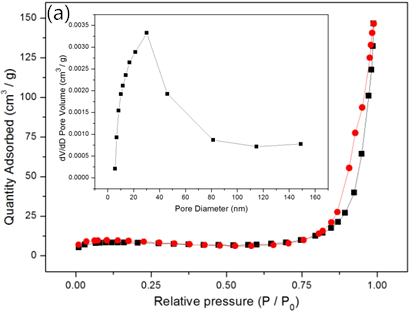

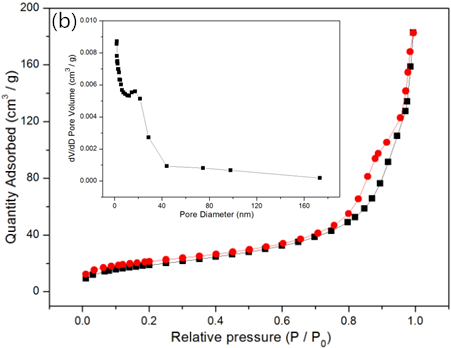


**Fig. S1** Nitrogen adsorption/desorption isotherm and Barrett-Joyner-Halenda (BJH) pore size distribution plot (inset) of the prepared hollow Fe_3_O_4_ precursor (a) and pristine NH_2_-MIL-101 (b).

**Fig. S2** Langmuir plots of the isotherm for phosphate adsorption to Fe_3_O_4_@NH_2_-MIL-101(Fe).

**Fig. S3** Plot of separation factor (R_L_) versus initial phosphate concentration to Fe_3_O_4_@NH_2_-MIL-101(Fe). **(**Fe_3_O_4_@NH_2_-MIL-101(Fe), 12mg; NaH_2_PO_4_ c_0_=0.1-1.0mgL^-1^; V=0.2L; T=293K).

**
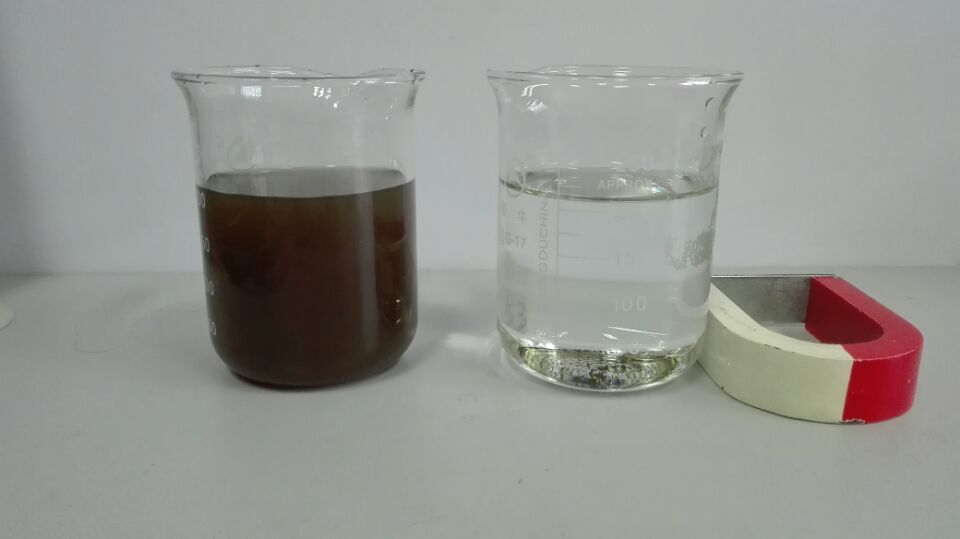
**

**Fig. S4** The fast magnetic collection of the disposals after adoption for 10 minutes.

**Thermodynamic study on the phosphate adsorption to Fe_3_O_4_**@**NH_2_-MIL-101(Fe)**

**Fig. S5** Effect of temperature on the phosphate **Fig. S6** Plot of lnKc vs. 1/T for the phosphate adsorption

adsorption to samples. to samples.

**(**Fe_3_O_4_@NH_2_-MIL-101(Fe), 12mg; NaH_2_PO_4_ c_0_=0.6mgL^-1^; V=0.2L)

The phosphate adsorption capacity of Fe_3_O_4_-NH_2_-MIL-101(Fe) is a little decreased with the increase of the adsorption temperature (Fig.S4). It shows that the adsorption process is an exothermic process, and the increase of temperature is not conducive to the phosphate adsorption by Fe_3_O_4_@NH_2_-MIL-101(Fe).In order to study the influence of temperature on the adsorption process and the phosphate adsorption mechanism of Fe_3_O_4_@NH_2_-MIL-101(Fe), the thermodynamic parameters of Gibbs free energyΔG^0^, Adsorption enthalpyΔH^0^, Adsorption entropyΔS^0^ were investigated.

Van 't Hoff equation: $\ln K_{c}=\frac{\Delta S^{0}}{R}-\frac{\Delta H^{0}}{RT}$ (1)

$\Delta G^{0}=-RT\ln K_{c}$ (2)

$K_{c}=\frac{q_{e}}{c_{e}}$ (3)

$\Delta G^{0}=\Delta H^{0}-T\Delta S^{0}$ (4)

According to Van 't Hoff equation, Plot of ln*K*_c_ vs. 1/T for the phosphate adsorption to Fe_3_O_4_@ NH_2_-MIL-101(Fe). (Fig. S5). It can be seen that ln*K*_c_ and 1/T basically showed a linear relationship (Correlation coefficients R^2^=0.95206). The adsorption thermodynamic equilibrium constant *K*_c_ decreased with the increase of temperature, which indicated that the low temperature was beneficial to adsorption. Thermodynamic parameters for the phosphate adsorption to Fe_3_O_4_@NH_2_-MIL-101(Fe) is listed in the following table 1.

**Table 1**. Thermodynamic parameters for the phosphate adsorption to Fe_3_O_4_@NH_2_-MIL-101(Fe)

| ***c*_0_/**  **mgL^-1^** | **T/**  **K** | ***K*_c_** | **ln*K*_c_** | **ΔG^0^/** $KJ\cdot\text{mol}^{-1}$ | **ΔH^0^/** $KJ\cdot\text{mol}^{-1}$ | **ΔS^0^/** $J\cdot\text{mol}^{-1}\cdot K^{-1}$ |
| --- | --- | --- | --- | --- | --- | --- |
| 0.6 | 283 | 187.35 | 5.23 | -12.31 | -9.78 | 9.13 |
|  | 293 | 175.58 | 5.17 | -12.59 |  |  |
|  | 303 | 139.53 | 4.94 | -12.44 |  |  |
|  | 313 | 126.14 | 4.84 | -12.60 |  |  |
|  | 323 | 116.67 | 4.76 | -12.78 |  |  |

In this study , Gibbs free energyΔG^0^ were in the range of -20~0$KJ\cdot\text{mol}^{-1}$, indicating that the adsorption process is mainly based on physical adsorption. Adsorption enthalpyΔH^0^(-9.78)<0, which further proved that the adsorption process was exothermic reaction, and the increase of temperature would be unfavorable to the phosphate adsorption of Fe_3_O_4_@NH_2_-MIL-101(Fe). The adsorption process of adsorption entropyΔS^0^ (9.13)>0, indicating the increase of the degree of confusion of the adsorbent and adsorbate in the solid-liquid interface, and the adsorption process is a process of entropy increase.

1. Corresponding author at: School of Chemistry Science and Engineering, Yunnan University, Kunming 650091, Yunnan, China. Tel. +86-871-65036626. E-mail address: guohongcom@126.com (Hong Guo), [yxjun@ynu.edu.cn](mailto:yxjun@ynu.edu.cn) (Xiangjun Yang) [↑](#footnote-ref-2)
